# Familial adversity: association with discontinuation of adjuvant hormone therapy and breast cancer prognosis

**DOI:** 10.1093/jnci/djae061

**Published:** 2024-03-12

**Authors:** Erwei Zeng, Wei He, Arvid Sjölander, Jenny Bergqvist, Fang Fang, Kamila Czene

**Affiliations:** Department of Medical Epidemiology and Biostatistics, Karolinska Institutet, Stockholm, Sweden; Department of Medical Epidemiology and Biostatistics, Karolinska Institutet, Stockholm, Sweden; Chronic Disease Research Institute, The Children’s Hospital, and National Clinical Research Center for Child Health, School of Public Health, School of Medicine, Zhejiang University, Hangzhou, Zhejiang, China; Department of Nutrition and Food Hygiene, School of Public Health, Zhejiang University, Hangzhou, Zhejiang, China; Department of Medical Epidemiology and Biostatistics, Karolinska Institutet, Stockholm, Sweden; Department of Medical Epidemiology and Biostatistics, Karolinska Institutet, Stockholm, Sweden; Institute of Environmental Medicine, Karolinska Institutet, Stockholm, Sweden; Department of Medical Epidemiology and Biostatistics, Karolinska Institutet, Stockholm, Sweden

## Abstract

**Background:**

Many studies have examined patient-related factors affecting adjuvant hormone therapy adherence in patients with breast cancer. Our study aimed to examine associations of family-related factors with adjuvant hormone therapy discontinuation and breast cancer–specific mortality.

**Methods:**

By cross-linking 7 Swedish health registers, we performed a cohort study that included all patients with breast cancer who initiated adjuvant hormone therapy during 2006-2019 in Sweden (N = 10 701). A group-based multitrajectory model was used to identify familial adversity groups based on 3 dimensions: material deprivation, negative family dynamics, and loss or threat of loss. Cox proportional hazard models were used to investigate associations of familial adversity with hormone therapy discontinuation and breast cancer–specific mortality.

**Results:**

We identified 5 distinctive familial adversity groups among the cohort participants. Compared with women who had low familial adversity, higher risks to discontinue adjuvant hormone therapy were observed among women with material deprivation (hazard ratio [HR] = 1.31, 95% confidence interval [CI] = 1.20 to 1.43), negative family dynamics (HR = 1.16, 95% CI = 1.06 to 1.28), loss or threat of loss (HR = 1.15, 95% CI = 1.00 to 1.32), or high familial adversity (HR = 1.53, 95% CI = 1.40 to 1.68). Furthermore, women with material deprivation (HR = 1.37, 95% CI = 1.05 to 1.79), negative family dynamics (HR = 1.41, 95% CI = 1.01 to 1.97), or high adversity (HR = 1.67, 95% CI = 1.26 to 2.23) were at higher risk of dying from breast cancer.

**Conclusion:**

Familial adversity is associated with a higher risk of adjuvant hormone therapy discontinuation and breast cancer–specific mortality. Family-related factors identified in our study may help identify high-risk patients for interventions to prevent treatment discontinuation and subsequently improve breast cancer outcomes.

Breast cancer is the most frequently diagnosed cancer in women, with approximately 2.3 million new cases diagnosed worldwide in 2020 (1). Approximately 79% to 84% of patients with breast cancer have estrogen receptor–positive cancer, and it is recommended that they receive adjuvant hormone therapy for 5 to 10 years to prevent recurrence and death from breast cancer (2-6). Many women discontinue adjuvant hormone therapy prematurely (7,8), however, which could lead to compromised survival benefits and increased medical costs (9-11).

The World Health Organization has highlighted that multiple factors influence medication adherence (12). Although great attention has been given to patient-related factors associated with discontinuation of adjuvant hormone therapy, the investigation of family-related factors is limited (8,13,14). Considering the family environment as a primary social setting for the development, maintenance, and modification of health behaviors, exploring family-related factors can provide additional insights into the reasons behind patients with breast cancer discontinuing their adjuvant hormone therapy (15).

Familial adversity, encompassing factors such as material deprivation (eg, family income, family member unemployment), negative family dynamics (eg, divorce, psychiatric illnesses, alcohol or drug abuse among family members), and loss or threat of loss of a significant other (eg, death or severe somatic illnesses of family members), is 1 such family-related factor that may contribute to adjuvant hormone therapy discontinuation (16-19). The potential impact of these familial adversities on hormone therapy discontinuation and breast cancer survival, however, remains unknown.

Linking data from 7 Swedish health registers, this population-based study aimed to investigate the association of familial adversity, including material deprivation, negative family dynamics, and loss or threat of loss, with adjuvant hormone therapy discontinuation and breast cancer–specific mortality.

## Methods

### Data source and study population

The Stockholm-Gotland Quality Register for Breast Cancer (the Stockholm-Gotland Breast Cancer Register, 1976-2007, and the Swedish National Quality Register for Breast Cancer, 2008 onward) collects information about tumor characteristics for all patients with breast cancer diagnosed in the Stockholm-Gotland region of Sweden (20,21). The Swedish Prescribed Drug Register has recorded dispensations of prescribed drugs in all Swedish pharmacies since July 2005, including data on each drug’s dispensation date, defined daily dose, days of supply, and classification code based on the Anatomic Therapeutic Chemical Classification System (22). The Prescribed Drug Register is highly complete, with less than 0.3% missing data (22). The longitudinal integrated database for health insurance and labour market studies (LISA) database has covered the Swedish population 16 years of age and older since 1990; it includes information about divorce, employment, and family income (23). Mandatory participation in the LISA database minimizes selection bias (23). The LISA variables used in this study exhibited high quality and have been widely used in previous research (23,24).

**Figure 1.**
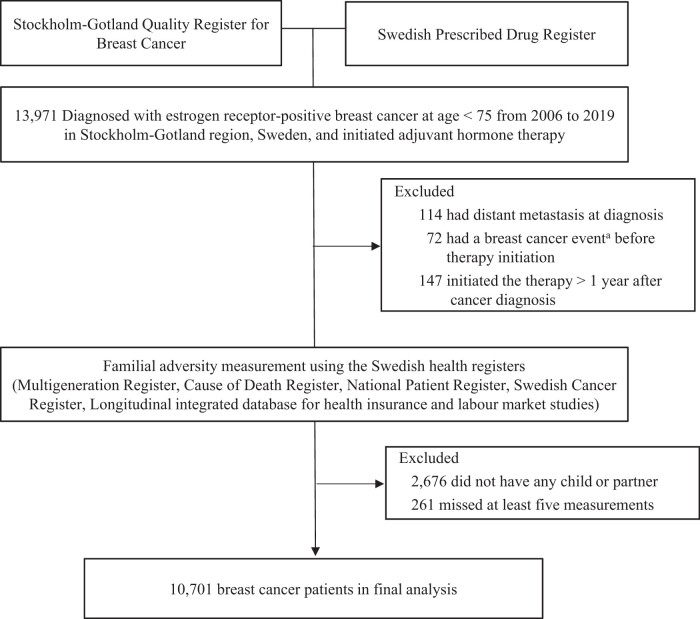
Flow chart of the study population. ^a^Breast cancer events include local recurrence, distant metastasis, and second primary breast cancer (>3 months after a primary breast cancer diagnosis).

The Stockholm-Gotland Quality Register for Breast Cancer and the Prescribed Drug Register were linked through unique Swedish personal identification numbers (25). Through this linkage, we identified 13 971 women diagnosed with estrogen receptor–positive breast cancer between 25 and 74 years of age during 2006-2019 in the Stockholm-Gotland region who initiated adjuvant hormone therapy with 1 or more prescriptions of tamoxifen (Anatomic Therapeutic Chemical code L02BA01) or aromatase inhibitors (Anatomic Therapeutic Chemical code L02BG). We excluded 114 patients with distant metastases at diagnosis, 72 patients who experienced a breast cancer event (ie, local recurrence, distant metastasis, or second primary breast cancer) before therapy initiation, 147 patients who initiated therapy more than 1 year after breast cancer diagnosis, 2676 patients with no children or partners (ie, biological father of the child), and 261 patients with missing data on more than 5 measurements of familial adversity, leaving 10 701 patients in the final cohort (Figure 1).

### Familial adversity

Through cross-linkages to the Swedish Multi-Generation Register, the LISA database, the Swedish Patient Register, the Swedish Cancer Register, and the Swedish Causes-of-Death Register, we linked the study participants to their children and partners to measure 13 familial adversity events, modified from a previous study (26). Given our focus on adulthood adversity, we replaced parents and siblings with partners and children, respectively, both of whom were core family members likely cohabiting with patients with breast cancer and directly influence the health behaviors of these patients. We also excluded foster care events as studied in the previous study due to data unavailability and its smaller relevance to adulthood adversity. Three predefined dimensions of familial adversity were used: material deprivation (ie, household-equivalized disposable income and partner unemployment), negative family dynamics (ie, divorce, psychiatric illnesses of a partner or child, and alcohol or drug abuse of a partner or a child), and loss or threat of loss (ie, death of a partner or child or severe somatic illnesses of a partner or child). Over a 10-year period before breast cancer diagnosis, all 13 familial adversity events were assessed for each year. Each adversity was allowed to occur once per year, and the same type of adversity could happen multiple times over the 10-year observation period. Each year, we calculated the total number of adversities within each dimension for every patient with breast cancer. Detailed information about the 13 familial adversity measures is provided in Supplementary Table 1 (available online).

A group-based multitrajectory model was used to identify distinctive groups of patients with breast cancer who followed similar familial adversity patterns across the 3 predefined dimensions (27). This model allowed us to incorporate complex longitudinal data and provided an efficient data summary to identify subgroups with similar trajectories of multiple exposures over time (27). We used the Stata package *TRAJ* (StataCorp LP, College Station, TX) to fit data with 1 to 8 familial adversity groups using zero-inflated Poisson regression with a cubic function of time to breast cancer diagnosis, yielding for each woman and familial adversity group the probability that the woman belonged to that group (27). We selected the model with 5 groups because most of the individuals had a very high probability (>90%) of belonging to a specific group under this model. Notably, instead of assuming the existence of a specific form of trajectories before, the model enabled the trajectory group to emerge naturally from the data. Based on the most distinctive feature in contrast to the other 4 groups, we labeled the 5 identified groups as “low adversity,” “material deprivation,” “negative family dynamics,” “loss or threat of loss,” and “high adversity.” The results from the group-based multitrajectory model may be biased if the modeling assumptions (ie, zero-inflated Poisson regression with a cubic function) are incorrect, but this caveat applies to virtually all model-based statistical inferences. The identified groups should be interpreted as a useful simplification of complex reality, representing clusters of individuals following approximately the same trajectory (27).

### Covariates

Information about age and calendar year of cancer diagnosis, tumor size, lymph node status, progestogen status, tumor grade, chemotherapy, and radiation therapy (RT) was retrieved from the Stockholm-Gotland Quality Register for Breast Cancer (20,21). The Charlson Comorbidity Index was calculated using all main diagnoses before breast cancer diagnosis in the Swedish Patient Register (28).

### Discontinuation of adjuvant hormone therapy

Discontinuation of adjuvant hormone therapy was defined as not refilling tamoxifen or aromatase inhibitors within 6 months after a previous prescription (7). Swedish pharmacies are allowed to dispense prescribed drugs for a maximum of 3 months. Therefore, not refilling tamoxifen or aromatase inhibitors for 6 months indicates missing at least 2 dispensations of adjuvant hormone therapy, suggesting treatment discontinuation. The study participants were followed from the first prescription of adjuvant hormone therapy until treatment discontinuation, local recurrence, distant metastasis, second primary breast cancer (>3 months after a primary breast cancer diagnosis), death, emigration, endometrial cancer diagnosis, venous thromboembolism diagnosis, the completion of 5 years of adjuvant hormone therapy, or the latest available date for drug dispensation data (August 31, 2020), whichever came first. Information about venous thromboembolism [*International Statistical Classification of Diseases, Tenth Revision (ICD-10)* codes I260, I269, I801, I808, I822, and I828] was retrieved from the Swedish Patient Register. Information about endometrial cancer (ICD-10 code C541) was retrieved from the Swedish Cancer Register.

### Breast cancer–specific mortality

Data on breast cancer–specific mortality were retrieved using the underlying cause of death through *ICD-10* code C50 in the Cause-of-Death Register. The cohort participants were followed up from the first prescription of adjuvant hormone therapy until death from breast cancer, death from other causes, emigration, or the latest available date for cause-of-death information (December 31, 2019), whichever came first.

### Statistical analyses

Kaplan-Meier curves were constructed to show the cumulative incidence of discontinuation of adjuvant hormone therapy by different familial adversity groups. A Cox proportional hazard model was used to investigate the association between familial adversity and the risk of adjuvant hormone therapy discontinuation. Wald tests were used to examine whether the association differed among familial adversity groups. We further investigated the association of familial adversity with breast cancer–specific mortality using Cox models. All Cox models were adjusted for age at diagnosis, calendar period of diagnosis, Charlson Comorbidity Index, tumor size, lymph node status, tumor grade, progestogen status, chemotherapy, and RT.

In the above analyses, each woman was allocated to the most probable familial adversity group. We conducted an additional analysis in which each woman instead contributed information to all familial adversity groups, weighted according to the estimated probability of belonging to each group. For instance, a woman who had a 90% probability of belonging to the “low adversity” group and a 10% probability of belonging to the “material deprivation” group was represented by 2 rows in the data set, 1 for each of these groups, which were given weights of 0.9 and 0.1, respectively, in the analysis. A robust sandwich covariance matrix estimate was used to account for the within-individual correlation. Additionally, we conducted a sensitivity analysis by using 5-year data before breast cancer diagnosis.

All analyses were performed using SAS, version 9.4 (SAS Institute, Inc, Cary, NC) and Stata, version 17.0, software. A 2-tailed ɑ level of .05 was used to determine statistical significance. The Regional Ethical Review Board in Stockholm, Sweden, approved the study. In the context of using Swedish register data, obtaining informed consent was not required.

## Results

**Figure 2.**
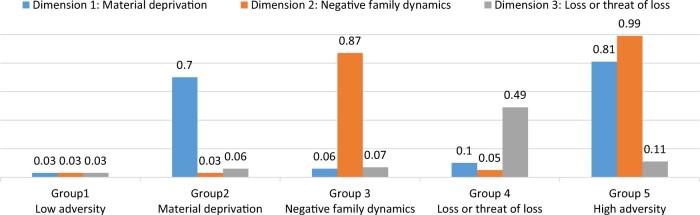
Average annual rate of family adversity over 10 years preceding breast cancer diagnosis in 3 dimensions, by familial adversity groups. Annual rate per person-year. Groups 1-5 were identified using the group-based multitrajectory model.

Figure 2 shows the 5 familial adversity groups identified among patients with breast cancer. Group 1, labeled “low adversity,” consisted of 44.3% of the patients and was characterized by a notably low level of familial adversity across all 3 dimensions. Groups 2, 3, and 4 were designated as “material deprivation” (18.5%), “negative family dynamics” (16.6%), and “loss or threat of loss” (6.5%), respectively. These groups exhibit high rates of material deprivation, negative family dynamics, and loss or threat of loss, aligning with their respective labels. A subset of patients, constituting 14.1%, fell into group 5, identified as “high adversity.” This group was characterized by elevated rates of both material deprivation and negative family dynamics, accompanied by a moderate rate of loss or threat of loss. Detailed family adversity trajectory over 10 years preceding cancer diagnosis, is shown in Supplementary Figure 1 (available online).

Table 1 shows the baseline characteristics of patients with breast cancer, categorized into different familial adversity groups. The material deprivation group had the highest proportion of lymph node–positive tumors and chemotherapy use. The proportion of patients with Charlson Comorbidity Index of 1 or higher was lowest in the low adversity group and highest in the high adversity group. A higher proportion of nonparticipation in breast cancer screening before cancer diagnosis was observed in women in the high adversity, material deprivation, and negative family dynamics groups than in the low adversity group.

**Table 1.** Baseline characteristics of Swedish women with estrogen receptor–positive breast cancer, by trajectory groups of familial adversity

Characteristics	Low adversity	Material deprivation	Negative family dynamics	Loss or threat of loss	High adversity	Difference test, *P*
Total No.	4740	1978	1781	696	1506	—
Age at diagnosis, median, y	59	58	62	66	61	<.001
Tumor size, No. (%)						.280
≤20 mm	3159 (66.6)	1288 (65.1)	1224 (68.7)	479 (68.8)	989 (65.7)	
>20 mm	1579 (33.3)	690 (34.9)	556 (31.2)	217 (31.2)	517 (34.3)	
Unknown	2 (0.0)	0 (0.0)	1 (0.1)	0 (0.0)	0 (0.0)	
Lymph node involvement, No. (%)						<.001
Negative	3266 (68.9)	1273 (64.4)	1243 (69.8)	501 (72.0)	1039 (69.0)	
Positive	1419 (29.9)	679 (34.3)	513 (28.8)	182 (26.1)	440 (29.2)	
Unknown	55 (1.2)	26 (1.3)	25 (1.4)	13 (1.9)	27 (1.8)	
Tumor grade, No. (%)						.421
1	973 (20.5)	402 (20.3)	415 (23.3)	146 (21.0)	314 (20.8)	
2	2636 (55.6)	1082 (54.7)	941 (52.8)	385 (55.3)	813 (54.0)	
3	1003 (21.2)	437 (22.1)	386 (21.7)	142 (20.4)	337 (22.4)	
Unknown	128 (2.7)	57 (2.9)	39 (2.2)	23 (3.3)	42 (2.8)	
Progesterone receptor status, No. (%)						.047
Positive	3999 (84.4)	1626 (82.2)	1480 (83.1)	571 (82.0)	1222 (81.1)	
Negative	737 (15.5)	348 (17.6)	300 (16.8)	124 (17.8)	284 (18.9)	
Unknown	4 (0.1)	4 (0.2)	1 (0.1)	1 (0.1)	0 (0.0)	
Chemotherapy, No. (%)						<.001
No	2522 (53.2)	1041 (52.6)	1016 (57.0)	412 (59.2)	878 (58.3)	
Yes	2198 (46.6)	933 (47.2)	756 (42.4)	282 (40.5)	624 (41.4)	
Unknown	20 (0.4)	4 (0.2)	9 (0.5)	2 (0.3)	4 (0.3)	
Radiation therapy, No. (%)						.014
No	570 (12.0)	269 (13.6)	208 (11.7)	90 (12.9)	236 (15.7)	
Yes	4157 (87.7)	1702 (86.0)	1568 (88.0)	605 (86.9)	1263 (83.9)	
Unknown	13 (0.3)	7 (0.4)	5 (0.3)	1 (0.1)	7 (0.5)	
Charlson Comorbidity Index, No. (%)						<.001
0	3937 (83.1)	1616 (81.7)	1417 (79.6)	538 (77.3)	1133 (75.2)	
1	482 (10.2)	209 (10.6)	224 (12.6)	84 (12.1)	212 (14.1)	
≥2	321 (6.8)	153 (7.7)	140 (7.9)	74 (10.6)	161 (10.7)	
Screening participation before diagnosis, No. (%)^a^						<.001
Nonparticipation	500 (12.4)	272 (17.8)	229 (14.3)	63 (10.5)	250 (19.7)	
Participation	3537 (87.6)	1253 (82.2)	1376 (85.7)	535 (89.5)	1022 (80.3)	

a Analysis for screening participation before diagnosis was restricted to patients diagnosed between ages 40 and 74 years (screening age) who received screening invitation.

### Familial adversity and discontinuation of adjuvant hormone therapy

**Figure 3.**
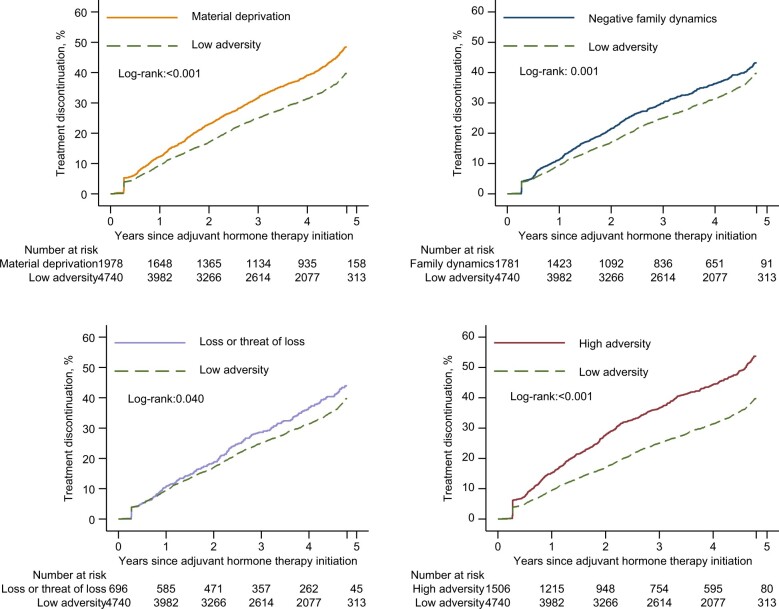
Discontinuation rate of adjuvant hormone therapy, by trajectory groups of familial adversity, in Swedish women with estrogen receptor–positive breast cancer.

During the 5-year follow-up, the cumulative incidence of discontinuation was 44.4%. The risk of discontinuation was lowest in the low adversity group (Figure 3). Compared with the low adversity group, the highest risk of discontinuation was observed in the high adversity group, with an adjusted hazard ratio (HR) of 1.53 (95% confidence interval [CI] = 1.40 to 1.68), followed by the material deprivation group (adjusted HR = 1.31, 95% CI = 1.20 to 1.43), the negative family dynamics group (adjusted HR = 1.16, 95% CI = 1.06 to 1.28), and the loss or threat of loss group (adjusted HR = 1.15, 95% CI = 1.00 to 1.32) (*P* < .001 for difference of hazard ratio) (Table 2). Analyses using the weighted Cox model showed similar results (Supplementary Table 2, available online).

**Table 2.** Association between familial adversity and discontinuation of adjuvant hormone therapy in Swedish women with estrogen receptor–positive breast cancer

Trajectory group	Total	Discontinuer	Incidence, per 1000 person-years	Hazard ratio (95% confidence interval)			
				Model 1^a^	*P*	Model 2^b^	*P*
Low adversity	4740	1492	99.2	1.00 (referent)	—	1.00 (referent)	—
Material deprivation	1978	839	131.0	1.32 (1.21 to 1.43)	<.001	1.31 (1.20 to 1.43)	<.001
Negative family dynamics	1781	593	116.0	1.18 (1.07 to 1.30)	.001	1.16 (1.06 to 1.28)	.002
Loss or threat of loss	696	240	113.8	1.15 (1.00 to 1.32)	.043	1.15 (1.00 to 1.32)	.044
High adversity	1506	691	154.8	1.58 (1.44 to 1.73)	<.001	1.53 (1.40 to 1.68)	<.001

a Model 1 adjusted for age at diagnosis and calendar period of diagnosis.

b Model 2 additionally adjusted for Charlson Comorbidity Index, tumor size, lymph node status, tumor grade, progestogen receptor status, chemotherapy, and radiation therapy.

### Familial adversity and breast cancer–specific mortality

Compared with those in the low adversity group, women with breast cancer were more likely to die from breast cancer if they were in the high adversity group (HR = 1.67, 95% CI = 1.26 to 2.23), material deprivation group (HR = 1.37, 95% CI = 1.05 to 1.79), or negative family dynamics group (HR = 1.41, 95% CI = 1.01 to 1.97), after adjusting for age at diagnosis, calendar period of diagnosis, tumor characteristics, and cancer treatment type (Table 3). Analyses using the weighted Cox model showed similar results (Supplementary Table 3, available online). Sensitivity analyses restricting familial adversity to 5 years before breast cancer diagnosis consistently demonstrated an association between the high adversity group and discontinuation of adjuvant hormone therapy (HR = 1.47, 95% CI = 1.34 to 1.61) as well as an association between the high adversity group and breast cancer mortality (HR = 1.74, 95% CI = 1.32 to 2.30). Because lymph node status is an important prognosticator of breast cancer mortality, sensitivity analyses to explore the association between family adversity and breast cancer mortality were conducted, stratified by lymph node status. The results revealed no statistically significant difference between the 2 subgroups (Supplementary Table 4, available online).

**Table 3.** Association between familial adversity and breast cancer mortality in Swedish women with estrogen receptor–positive breast cancer

Trajectory group	Total^a^	Death from breast cancer	Incidence, per 1000 person-years	Hazard ratio (95% confidence interval)			
				Model 1^b^	*P*	Model 2^c^	*P*
Low adversity	4615	116	4.3	1.00 (referent)		1.00 (referent)	
Material deprivation	1947	107	7.0	1.47 (1.13 to 1.91)	.005	1.37 (1.05 to 1.79)	.019
Negative family dynamics	1719	51	5.8	1.44 (1.03 to 2.00)	.032	1.41 (1.01 to 1.97)	.041
Loss or threat of loss	689	21	5.6	1.33 (0.83 to 2.12)	.236	1.25 (0.78 to 2.00)	.352
High adversity	1487	82	8.1	1.83 (1.37 to 2.43)	<.001	1.67 (1.26 to 2.23)	<.001

a The analysis excluded patients who initiated adjuvant hormone therapy after December 31, 2019, because cause-of-death information is available only up to that date.

b Model 1 adjusted for age at diagnosis and calendar period of diagnosis.

c Model 2 additionally adjusted for Charlson Comorbidity Index, tumor size, lymph node status, tumor grade, progestogen receptor status, chemotherapy, and radiation therapy.

## Discussion

Our population-based study showed that more than half of all patients with breast cancer experienced some degree of familial adversity over the decade before diagnosis. Familial adversity could take the form of material deprivation, negative family dynamics, and loss or threat of loss in the family. Patients who were exposed to some level of familial adversity before diagnosis were at a higher risk of adjuvant hormone therapy discontinuation than patients who were exposed to a low level of familial adversity. The risk was most pronounced in women who were exposed to a moderate to high level of familial adversity in all 3 dimensions. Notably, the women who experienced familial adversity were also more likely to die from breast cancer.

Patients with breast cancer often receive decreased support from health-care professionals after completion of their hospital-administered treatments (29). Consequently, the role of family members becomes vital in encouraging patients to adhere to the long-term use of adjuvant hormone therapy thereafter (30). Our study provides a new perspective by exploring the significance of family-related factors—namely, familial adversity—in relation to adjuvant hormone therapy discontinuation. This perspective complements existing research on individual risk factors for treatment discontinuation.

Our results are in line with previous research showing that specific familial adversities, such as divorce, loss of spouse, and low family income, were associated with discontinuation of adjuvant hormone therapy (16-18,31-33). In contrast to prior studies that relied on a single measurement, our study incorporated repeated measurements of familial adversity during the 10 years before diagnosis. Additionally, our study contributes to the existing literature by employing a novel methodology that incorporates multiple dimensions of familial adversity, including material deprivation, negative family dynamics, and loss or threat of loss. This method enabled us to examine their joint effect on treatment discontinuation. Our study found a subgroup experiencing all 3 adversities simultaneously, exhibiting the most pronounced negative impact on treatment discontinuation compared with the experience of any single adversity. These findings underscore the need for additional follow-up and greater support for patients with breast cancer living in vulnerable families to prevent treatment discontinuation.

Several mechanisms can explain the association between familial adversity and adjuvant hormone therapy discontinuation. One potential explanation is that patients with breast cancer who experience familial adversity may face difficulties in receiving essential support from family members, potentially affecting their motivation to adhere to the treatment (34). Another explanation is that experiencing adverse familial events increases psychological distress. The emotional strain resulting from familial adversity, such as financial strain, bereavement, or negative family dynamics (eg, a partner being diagnosed with drug or alcohol abuse), has been shown to increase levels of psychological burden, which can impede women's ability to manage treatment-related symptoms and adhere to treatment (35-37). The complex interplay between familial adversity and treatment discontinuation warrants further investigation.

There is a noteworthy impact of postdiagnosis familial adversity on hormone therapy discontinuation. Our analysis indicated a statistically significant moderate correlation between prediagnosis and postdiagnosis familial adversity (data not shown). Studying postdiagnosis familial adversity is challenging, however, because of the necessity of examining familial adversity after breast cancer diagnosis but before therapy discontinuation or censoring events during the follow-up, leading to variations in measurement time points among patients with different follow-up times. Challenges also arise from the intricate interplay between breast cancer diagnosis and postdiagnosis familial adversity.

Our study also revealed that patients with breast cancer who experience familial adversity were at a higher risk of dying from breast cancer, which may be partially explained by the observed higher risk of adjuvant hormone therapy discontinuation among these patients. It is important to recognize, however, that the variations in breast cancer mortality among individuals with different levels of familial adversity can be attributed to other factors. For instance, our study observed a higher nonparticipation rate in breast cancer screening among women experiencing familial adversity, which could result in delayed diagnosis and thus a negative impact on survival. Moreover, a higher proportion of women with lymph node–positive disease was observed in the material deprivation group, potentially elucidating their elevated breast cancer mortality. Even after restricting the analysis to women without positive lymph nodes, however, a statistically significant association between material deprivation and mortality persisted. Additionally, factors such as disparities in access to health care and lifestyle factors may contribute to the differences in breast cancer mortality (38-41).

Our study had several strengths. The linkage of data in health registers enabled us to identify family members and ascertain family information. In addition, it enabled us to incorporate data on familial adversity from a decade before breast cancer diagnosis. Several limitations also need to be acknowledged. First, the discontinuation rate of adjuvant hormone therapy might have been underestimated in the study sample because some patients might not have taken the medication, despite it being dispensed. Second, register information may not capture all adverse events that a woman might experience within the family, such as domestic violence, interpersonal conflicts, and crimes committed by family members. Additionally, despite using data on hospital visits related to drug or alcohol abuse and psychiatric illness, these events of family members might not be completely captured if they were not presented to the health care professional. As a result, the register-based definition of *familial adversity* might be an underestimate of the real familial adversity and its true effect on outcomes. Third, we were unable to investigate whether the increased breast cancer mortality rate in higher familial adversity groups was explained by nonadherence to other breast cancer treatments due to lack of data on chemotherapy and RT adherence. The majority of patients with breast cancer in Sweden adhere to chemotherapy and RT, however, unless they experience severe treatment-related side effects (42).

Our study provides a novel perspective on the role of family-related factors—namely, familial adversity—on adjuvant hormone therapy discontinuation in patients with breast cancer. Evaluating family information of patients with breast cancer may help identify groups at risk of treatment discontinuation and therefore improve treatment outcome and breast cancer survival.

## Supplementary Material

djae061_Supplementary_Data

## Data Availability

The data underlying this article were provided by the National Quality Register for Breast Cancer (https://skr.se/en/kvalitetsregister/hittaregister/registerarkiv/brostcancer.44178.html), Socialstyrelsen (https://www.socialstyrelsen.se/), and Statistics Sweden (https://www.scb.se/en/) by permission. Data are not allowed to be shared due to Swedish laws, but it is possible to apply for the same data for research from Swedish registers.
