# Supplementary material for: Familial adversity: association with discontinuation of adjuvant hormone therapy and breast cancer prognosis
Source: J Natl Cancer Inst. 2024 Mar 12;116(6):920–8. doi: 10.1093/jnci/djae061 (PMC11160492; doi:10.1093/jnci/djae061)
Supplement: djae061_Supplementary_Data [file djae061_supplementary_data.docx]

**Familial adversity: association with discontinuation of adjuvant hormone therapy and breast cancer prognosis**

**Supplementary**

**Supplementary Table 1.** Dimensions and definitions of familial adversity events

**Supplementary Table 2.** Association between familial adversity and discontinuation of adjuvant hormone therapy in Swedish women with ER-positive breast cancer using weight Cox model

**Supplementary Table 3.** Association between familial adversity and breast cancer mortality in Swedish women with ER-positive breast cancer using weight Cox model

**Supplementary Table 4.** Association between familial adversity and breast cancer mortality in Swedish women with ER-positive breast cancer, stratified by lymph node status

**Supplementary Figure 1.** Estimated trajectory groups of familial adversity among Swedish women with ER-positive breast cancer, 2006-2019

**Supplementary Table 1.** Dimensions and definitions of familial adversity events

| **Dimension** | **Definition** | **Registers** |
| --- | --- | --- |
| **Material deprivation** | | |
| Low family income | Household-equivalised disposable income in the lowest quintile within the strata of similar working age (i.e., <20/20-44/45-64/65+) at year of measurement | The Longitudinal integrated database for health insurance and labour market studies |
| Partner’s unemployment | Unemployment of a partner | The Longitudinal integrated database for health insurance and labour market studies |
| **Negative family dynamics** | | |
| Divorce | Being divorced | The Longitudinal integrated database for health insurance and labour market studies |
| Partner’s psychiatric illness | A partner being diagnosed one of the psychiatric illnesses, excluding main diagnoses related to alcohol and drug abuse (ICD-9/ICD-10 codes: 293-319 except 303 and 304/F00–F99 except F10–F19) | The Swedish National Patient Register |
| Child’s psychiatric illness | A child being diagnosed one of the psychiatric illnesses, excluding main diagnoses related to alcohol and drug abuse (ICD-9/ICD-10 codes: 293-319 except 303 and 304/F00–F99 except F10–F19) | The Swedish National Patient Register |
| Partner’s alcohol abuse | A partner being diagnosed with an illness related to alcohol abuse (ICD-9/ICD-10 codes): alcohol psychosis and abuse syndrome (291, 303/F10); alcoholic polyneuropathy (357F/G621); alcoholic cardiomyopathy (425F/I426); alcoholic-induced acute (K852) and chronic (577B/K860) pancreatitis; alcoholic liver disease (571A-D/K70); alcoholic gastritis (535D/K292). | The Swedish National Patient Register |
| Partner’s drug abuse | A partner being diagnosed with an illness related to drug abuse (ICD-9/ICD-10 codes: 292, 304/ F11-19 except F17). | The Swedish National Patient Register |
| Child’s alcohol abuse | A child being diagnosed with an illness related to alcohol abuse (ICD-9/ICD-10 codes): alcohol psychosis and abuse syndrome (291, 303/F10); alcoholic polyneuropathy (357F/G621); alcoholic cardiomyopathy (425F/I426); alcoholic-induced acute (K852) and chronic (577B/K860) pancreatitis; alcoholic liver disease (571A-D/K70); alcoholic gastritis (535D/K292). | The Swedish National Patient Register |
| Child’s drug abuse | A child being diagnosed with an illness related to drug abuse (ICD-9/ICD-10 codes: 292, 304/ F11-19 except F17). | The Swedish National Patient Register |
| **Loss or threat of loss** | | |
| Death of partner | Death of a partner | The Swedish Cause of Death Register |
| Death of child | Death of a child | The Swedish Cause of Death Register |
| Partner’s somatic illness | A partner being diagnosed with one of the illnesses included in the Charlson Comorbidity Index | The Swedish National Patient Register; The Swedish Cancer Register |
| Child’s somatic illness | A child being diagnosed with one of the illnesses included in the Charlson Comorbidity Index  If a child is under 18 years old in the given year, one count per year of life for a child diagnosed with one of the seven most common somatic illnesses related to mortality in children aged 0–18 years (ICD-9/ICD-10 codes): malignant neoplasm (140–209/C00–C96); congenital anomalies of the heart and circulatory system (745-747, /Q20–Q28); congenital anomalies of the nervous system (740-741/Q00–Q07); cerebral palsy (342-344/G80–G83); epilepsy (333C, 345/G40–G41); cardiomyopathy (425/I42–I43); congenital disorders of lipid metabolism (272C, 272H, 330A, 330B/E75) | The Swedish National Patient Register; The Swedish Cancer Register |

**Supplementary Table 2.** Association between familial adversity and discontinuation of adjuvant hormone therapy in Swedish women with ER-positive breast cancer using weight Cox model

|  |  |  | Incidence, per 1000 person-years | HR (95% CI) ^a^ | | | |
| --- | --- | --- | --- | --- | --- | --- | --- |
| Trajectory group | Total | Discontinuer |  | Model 1^b^ | *p-value* | Model 2^c^ | *p-value* |
| Low adversity | 4657.3 | 1465.6 | 99.3 | 1.00(reference) |  | 1.00(reference) |  |
| Material deprivation | 1985.8 | 837.5 | 130.2 | 1.31(1.20-1.42) | <0.001 | 1.30(1.20-1.41) | <0.001 |
| Family dynamics | 1775.1 | 591.8 | 116.1 | 1.18(1.08-1.29) | <0.001 | 1.17(1.07-1.28) | <0.001 |
| Loss or threat of loss | 754.0 | 259.2 | 112.2 | 1.13(1.01-1.27) | 0.036 | 1.13(1.00-1.27) | 0.044 |
| High adversity | 1528.8 | 700.9 | 154.7 | 1.58(1.44-1.72) | <0.001 | 1.55(1.42-1.69) | <0.001 |

^a^ HRs were estimated using Cox proportional hazard model weighted by the probability of patients belonging to each familial adversity group.

^b^ Model 1 adjusted for age at diagnosis and calendar period of diagnosis.

^c^ Model 2 additionally adjusted for Charlson Comorbidity Index, tumor size, lymph node status, tumor grade, progestogen receptor status, chemotherapy and radiotherapy.

**Supplementary Table 3.** Association between familial adversity and breast cancer mortality in Swedish women with ER-positive breast cancer using weight Cox model

|  |  | Death from breast cancer | Incidence, per 1000 person-years | HR (95% CI) ^a^ | | | |
| --- | --- | --- | --- | --- | --- | --- | --- |
| Trajectory group | Total |  |  | Model 1^b^ | *p-value* | Model 2^c^ | *p-value* |
| Low adversity | 4533.6 | 115.0 | 4.4 | 1.00(reference) |  | 1.00(reference) |  |
| Material deprivation | 1954.7 | 104.2 | 6.9 | 1.41(1.09-1.82) | 0.008 | 1.34(1.03-1.73) | 0.027 |
| Family dynamics | 1715.5 | 51.7 | 5.9 | 1.44(1.05-1.96) | 0.023 | 1.40(1.03-1.92) | 0.033 |
| Loss or threat of loss | 745.0 | 22.2 | 5.3 | 1.22(0.82-1.82) | 0.326 | 1.17(0.78-1.76) | 0.448 |
| High adversity | 1508.1 | 83.9 | 8.2 | 1.82(1.38-2.39) | <0.001 | 1.69(1.27-2.23) | <0.001 |

^a^ HRs were estimated using Cox proportional hazard model weighted by the probability of patients belonging to each familial adversity group.

^b^ Model 1 adjusted for age at diagnosis and calendar period of diagnosis.

^c^ Model 2 additionally adjusted for Charlson Comorbidity Index, tumor size, lymph node status, tumor grade, progestogen receptor status, chemotherapy and radiotherapy.

Supplementary Table 4. Association between familial adversity and breast cancer mortality in Swedish women with ER-positive breast cancer, stratified by lymph node status

|  |  | | Death from breast cancer | Incidence, per 1000 person-years | HR (95% CI) ^a^ | | | |
| --- | --- | --- | --- | --- | --- | --- | --- | --- |
| Trajectory group | | Total |  |  | Model 1^a^ | *p-value* | Model 2 ^b^ | *p-value* |
| **Lymph node negative** | |  |  |  |  |  |  |  |
| Low adversity | | 3200 | 38 | 2.0 | 1.00(reference) |  | 1.00(reference) |  |
| Material deprivation | | 1254 | 36 | 3.6 | 1.58(1.00-2.50) | 0.052 | 1.67(1.05-2.64) | 0.030 |
| Family dynamics | | 1204 | 13 | 2.1 | 1.14(0.61-2.15) | 0.684 | 1.17(0.62-2.21) | 0.632 |
| Loss or threat of loss | | 498 | 9 | 3.3 | 1.74(0.84-3.63) | 0.137 | 1.62(0.78-3.38) | 0.197 |
| High adversity | | 1027 | 34 | 4.9 | 2.29(1.44-3.65) | 0.001 | 2.27(1.42-3.62) | 0.001 |
| **Lymph node positive** | |  |  |  |  | . |  | . |
| Low adversity | | 1369 | 76 | 9.5 | 1.00(reference) | . | 1.00(reference) | . |
| Material deprivation | | 668 | 71 | 13.9 | 1.37(0.99-1.90) | 0.059 | 1.30(0.93-1.80) | 0.123 |
| Family dynamics | | 494 | 37 | 14.1 | 1.49(1.00-2.21) | 0.048 | 1.51(1.01-2.24) | 0.043 |
| Loss or threat of loss | | 178 | 11 | 10.8 | 1.08(0.57-2.05) | 0.802 | 1.09(0.58-2.07) | 0.786 |
| High adversity | | 436 | 47 | 15.5 | 1.59(1.10-2.30) | 0.013 | 1.41(0.97-2.03) | 0.072 |

^a^ Model 1 adjusted for age at diagnosis and calendar period of diagnosis.

^b^ Model 2 additionally adjusted for Charlson Comorbidity Index, tumor size, lymph node status, tumor grade, progestogen receptor status, chemotherapy and radiotherapy.

**Supplementary Figure 1.** Estimated trajectory groups of familial adversity over ten years preceding breast cancer diagnosis among Swedish women with ER-positive breast cancer, 2006-2019


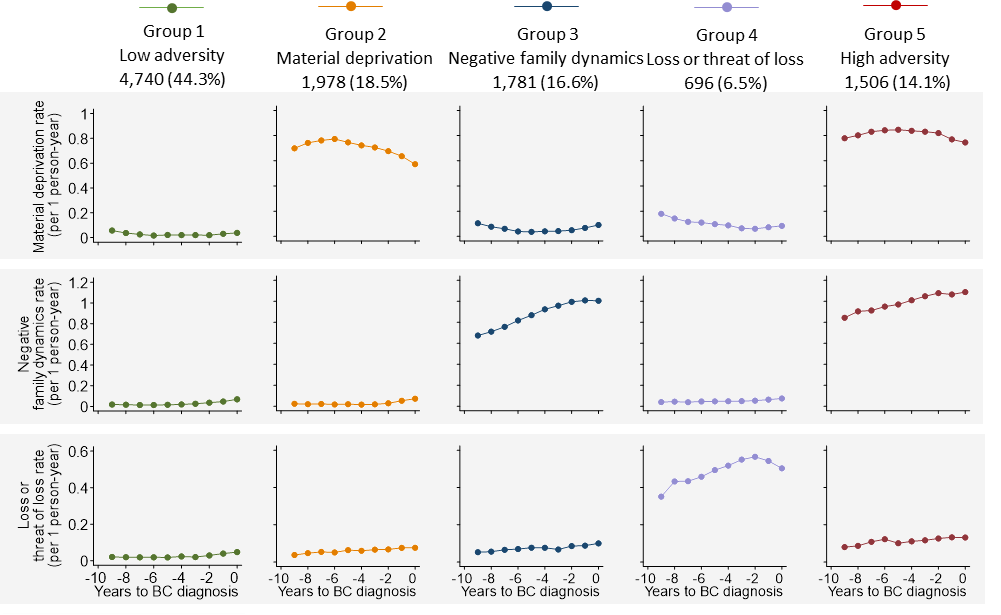


Note. Group 1-5 were identified via the group-based multi-trajectory model.
